# A Novel Fisheye-Lens-Based Photoacoustic System

**DOI:** 10.3390/s16122185

**Published:** 2016-12-19

**Authors:** Hojong Choi, Jaemyung Ryu, Jungsuk Kim

**Affiliations:** 1 Department of Medical IT Convergence Engineering, Kumoh National Institute of Technology, Gumi 39253, Korea; hojongch@kumoh.ac.kr; 2 Department of Optical System Engineering, Kumoh National Institute of Technology, Gumi 39253, Korea; jmryu@kumoh.ac.kr; 3 Department of Biomedical Engineering, Gachon University, In-cheon 21936, Korea

**Keywords:** fisheye lens, photoacoustic, ultrasound, transducers

## Abstract

This paper presents a novel fisheye-lens-based photoacoustic (PA) system. In conventional PA systems, mechanical motors are utilized to obtain the target information due to the small fields of view of such systems. The use of such motors introduces mechanical noise, which is difficult to remove when processing the echo signals. A fisheye lens system offering a wide field of view would effectively reduce the motor effects (i.e., the noise) and enable the system to have a wide field of view. Therefore, in this work, we propose a novel fisheye lens scheme and describe a PA system based on the developed lens scheme. In addition, to confirm the feasibility of the fisheye-lens-based PA system, we present the typical pulse-echo responses obtained using a 20 MHz single element immersion transducer and the echo signals measured from bull’s eye tissue samples separated by approximately 4, 6, 8, and 10 cm diagonally and 2 cm vertically from the fisheye lens. The experimental results demonstrate that the echo signal amplitudes, their center frequencies, and the −6 dB bandwidths obtained using red, green, and blue lights and a fisheye lens are acceptable when the fisheye lens is separated from a sample both diagonally and vertically. Therefore, fisheye-lens-based PA systems could be a potential method of achieving wide fields of view while reducing the mechanical motor effects.

## 1. Introduction

Photoacoustic (PA) systems have recently been developed to obtain anatomical and structural information [1]. In a PA system, discrete or continuous light pulses are used to obtain the target information [2], the energy absorbed from the light generated by the target produces a transient thermal expansion, and the increased thermal emission energy is then detected by an ultrasound transducer to determine the target’s biological information [3]. Optical wavelengths between 550 nm and 900 nm are typically used in PA systems, and the operating range is determined based on the type of tissue, such as hemoglobin, melanin, or water, whose information needs to be obtained [3,4]. Compared to conventional optical systems, PA systems yield much weaker ultrasonic signals from biological targets because of the scattering of the reflected ultrasound waves [3,5,6].

Photoacoustic approaches can be divided into two major categories: photoacoustic tomography methods and photoacoustic microscopy methods [7]. Photoacoustic tomography techniques are more traditional and are used to diffuse pulsed light to enable it to penetrate deeply and to be scattered throughout large areas [4]. The light is detected by mechanical scanning transducers, and receiver electronics and reconstruction algorithms are employed to map the target information [3]. In photoacoustic microscopy methods, focused beams or transducers with mechanical scanning motors are used to achieve high sensitivity [3]. If a focused transducer is used, the method is called acoustic resolution photoacoustic microscopy (AR-PAM) because the spatial resolution is defined by the characteristics of the ultrasonic transducer [7]. An AR-PAM system typically includes a single mechanical scanning focused transducer [3]. To detect an acoustic signal in two dimensions, the light beam or the transducer need to be scanned by a motor [8]. However, an array-type transducer could also be used to scan the target information via the electronic scanning method. If a focused beam is used, the technique is called optical resolution photoacoustic microscopy (OR-PAM) because the spatial resolution is primarily affected by the light beam [7]. A highly focused optical lens is used to focus the light beam on the target [7]. The mechanical motor supports the light beam and ultrasonic transducer to enable the echo signal amplitude to be detected in the pulse-echo A-mode [3]. Compared to AR-PAM, OR-PAM yields higher lateral resolutions of up to a few microns but reduced penetration depths since the optical wavelengths are shorter [7]. Using an x-y galvanometer scanner to scan the focused light beam could avoid the adverse effects of mechanical scanning but would reduce the field of view and cause optical lens aberrations [7]. Therefore, these PA methods are used to obtain target information by employing mechanical motors to cover wide areas [9,10]. However, a motor typically generates mechanical noise, which directly decreases the echo signal quality [6,10,11]. It is thus imperative to be able to obtain information from wide areas without using motors. Employing a fisheye lens offering a wide field of view with an electrical signal could be an effective means of achieving wide-angle views of targets. Fisheye lenses have been widely adopted in electronic switching systems for security cameras, virtual tours, and surveillance applications that operate in the visible and infrared wavelength ranges [12,13,14]. Such lenses can also be employed in PA systems when light with a wavelength between 550 nm and 900 nm is used because of the similarity between the wavelength requirements of these applications. Thus, in this report, we will propose a novel fisheye lens scheme, describe a PA system based on the developed lens scheme, and demonstrate its performance experimentally. A light beam passing through a fisheye lens can cover a wide field of view [15]. Thus, if a fisheye lens is employed in a PA system, the mechanical motor effects can be reduced, decreasing the mechanical noise and consequently increasing the quality of the echo signal that the PA system receives. To the best of our knowledge, fisheye lenses have never before been applied in PA systems. The main objective of this research was to design an optical lens system with a wide field of view and thereby to determine the feasibility of reducing mechanical noise by using the developed lens system and ultrasonic transducers. Therefore, our first priority was to prove our concept experimentally rather than to obtain images using the PA system. Thus, we designed and fabricated a fisheye lens system and developed a PA system using this lens system. To prove the effectiveness of our fisheye-lens-based PA system, we employed a typical A-mode pulse-echo method, which is one of gold standards for checking developed acoustic systems.

In general, the first step in designing a lens scheme is to choose the structure of each lens in the entire scheme. An initial structure is designed to determine the refracting power of each lens group based on paraxial theory and to minimize the third-order aberrations resulting from the curvature at each lens surface [16]. In this study, we designed a fisheye lens scheme suitable for use in our PA system since fisheye lens schemes enable target information to be obtained easily, even when the location of the light source or target changes. An additional advantage of our fisheye lens system over conventional fisheye lens systems is that only one lens is moved to focus the light in this case. Generally, fisheye lens systems have wide fields of view but large first lenses, making it impossible to fabricate compact PA systems with electrical switches.

**Figure 1:**
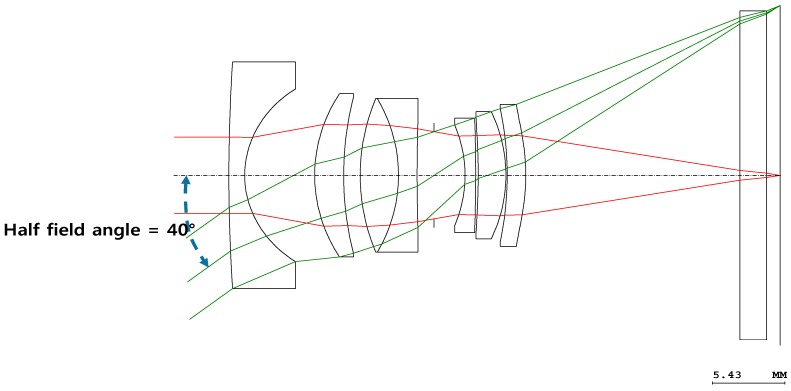
Optical layout of a conventional wide-angle system.

Figure 1 illustrates the optical paths in a conventional wide-angle system that has a half-field angle of 40°. A conventional wide-angle system (fisheye lens system) has a wide field of view and should be of the retro-focus type [16]. In a retro-focus system, the front lens group has a negative refracting power [17]. Therefore, the back focal length (BFL) of the lens group is greater than its effective focal length (EFL) [17].

## 2. Methods

**Figure 2:**
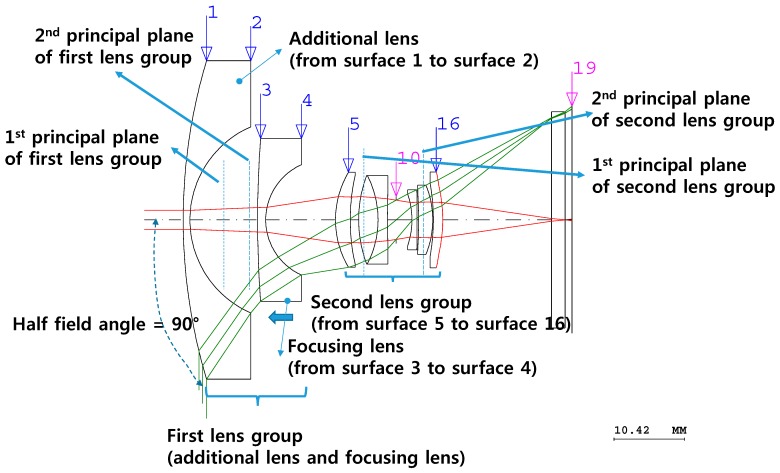
Optical layout of the designed fisheye lens for the paraxial design.

Figure 2 depicts the designed paraxial fisheye lens system after adding one additional lens. The one additional lens is necessary to achieve a half-field angle of 90°. Like the fisheye lens scheme in Figure 2, this fisheye lens scheme with a half-field angle of 90° needs to have a shorter EFL. However, the paraxial design does not yield a suitable optical resolution because the lens system minimizes only third-order aberrations. Therefore, the system must be optimized to improve the optical resolution for the modulation transfer function (MTF).

In the fisheye lens system shown in Figure 2, one additional lens is used to increase the half-field angle to 90°, and the subsequent lens (the focusing lens in Figure 2) is employed to compensate for changes in the image plane due to changes in the object distance. In such a lens system, the focusing lens works as an inner-focus-type lens, i.e., it does not change the total distance of the lens when focusing the image. The types of lenses used between surface 3 and the last surface (surface 19) are identical to those in the conventional wide-angle system shown in Figure 1.

As illustrated in Figure 2, an additional lens is included between surfaces 1 and 2, and the subsequent lens (the focusing lens in Figure 2) is for focusing. Therefore, the first lens group includes the additional lens and the subsequent focusing lens, and the second lens group is located between surfaces 5 and 16. The flat piece of glass in front of the image plane (surface 19) works as an infrared-cut filter to protect the digital image sensor and reduce the thermal noise. Flat glass usually minimally affects fisheye lens systems, which have large *f*-numbers (F/#). Therefore, the relationship between the height of the axial ray at the image plane and the EFL of the fisheye lens system is as represented in Equation (1) using Gaussian brackets [17]:

(1) $$\begin{array}{l} \left[k_{1},-z_{1},k_{2}\right]=k_{1}+k_{2}-z_{1}k_{1}k_{2}=K\\ \left[k_{1},-z_{1},k_{2},-z_{2}\right]=-z_{2}\left(k_{1}+k_{2}-z_{1}k_{1}k_{2}\right)+1-k_{1}z_{1}=-z_{2}K+1-k_{1}z_{1}=0 \end{array}$$

where *k*_1_ and *k*_2_ are the refracting powers of the first and second lens groups, respectively; *z*_1_ is the distance between the second principal plane of the first lens group and the first principal plane of the second lens group; *z*_2_ is the distance from the second principal plane of the second lens group to the image plane; and *K* is the refracting power of the entire fisheye lens system and equals the reciprocal of the EFL.

In the paraxial system, the refracting power *k*_2_ is known since one additional lens is added to the conventional fisheye lens system, and *z*_2_ is also known since the additional lens is designed not to change the BFL. *K* is 1/10.1 mm^−1^ if the focal length of the designed fisheye lens system is assumed to be 10.1 mm. If *k*_2_ and *z*_1_ remain unknown, Equation (1) becomes:

(2) $$f_{1}=\frac{F}{1-z_{2}k_{2}}=\frac{F}{1-\frac{z_{2}}{f_{2}}},\;z_{1}=\left(1-\frac{z_{2}}{F}\right)f_{1}=\frac{F\cdot \left(1-\frac{z_{2}}{F}\right)}{1-\frac{z_{2}}{f_{2}}}$$

where, *f*_1_ is the focal length of the first lens group and *f*_1_, *f*_2_, and *F* are the reciprocals of *k*_1_, *k*_2_, and *K*, respectively.

By employing Equation (2), the shape of the additional lens in Figure 2 can be determined. If the focal length of the first lens group is known, *F* can be calculated using Equation (2). *f*_2_ can also be determined if the focal length of focusing lens between surfaces 3 and 4 is known and if it is the same as that of the focusing lens in the paraxial system shown in Figure 2. The BFL of the first lens group is negative since the focal length is negative, so *z*_2_ can also be determined. A lens thickness of 1 mm was selected in this study, considering the machinability of the lens. BSC7 (Hoya Corporation) was chosen as the lens material since it has a low refractive index and relatively low cost [18]. Therefore, the focal length of the additional lens could be determined. However, third-order aberrations in this kind of fisheye lens system are not well compensated for, so the numbers of the aspheric plane of surface 16 needed to be adjusted.

The shape of the aspheric plane in Figure 2 can be represented by a curvature, a conic constant *k*, and the fourth coefficient *A*_4_, which are related to the third-order aberrations as follows:

(3) $$\begin{array}{l} z(r)=\frac{cr^{2}}{1+\sqrt{1-(1+k)c^{2}r^{2}}}+A_{4}r^{4}+A_{6}r^{6}+A_{8}r^{8}+A_{10}r^{10}+\Lambda \\ r^{2}\equiv x^{2}+y^{2} \end{array}$$

where *c* is the curvature of surface 16 and *x* and *y* are the coordinates of a random point.

The expressions for the contributions of the third-order aberrations due to the aspheric surface are given by [19]:

(4) $$\begin{array}{l} S_{I}^{*}=\left(kc^{3}+8A_{4}\right)\left(n_{i-1}-n_{i}\right)h_{i}^{4}\\ S_{II}^{*}=\left(kc^{3}+8A_{4}\right)\left(n_{i-1}-n_{i}\right)h_{i}^{3}{\bar{h}}_{i}\\ S_{III}^{*}=\left(kc^{3}+8A_{4}\right)\left(n_{i-1}-n_{i}\right)h_{i}^{2}{\bar{h}}_{i}^{2}\\ S_{IV}^{*}=0\\ S_{V}^{*}=\left(kc^{3}+8A_{4}\right)\left(n_{i-1}-n_{i}\right)h_{i}{\bar{h}}_{i}^{3} \end{array}$$

where *n* is the refractive index, *h* is the height of the axial ray, $\bar{h}$ is the height of the principal ray, and *i* is the number of the surface and equals 16 since the aspheric surface is surface 16. *k* and *A*_4_ can be adjusted to eliminate these contributions and obtain a fisheye lens system in which the third-order aberrations are compensated for.

**Figure 3:**
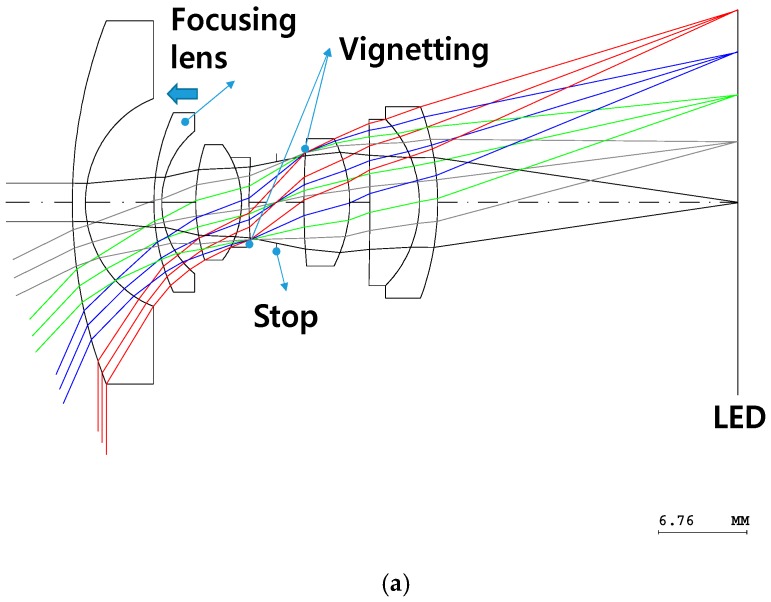

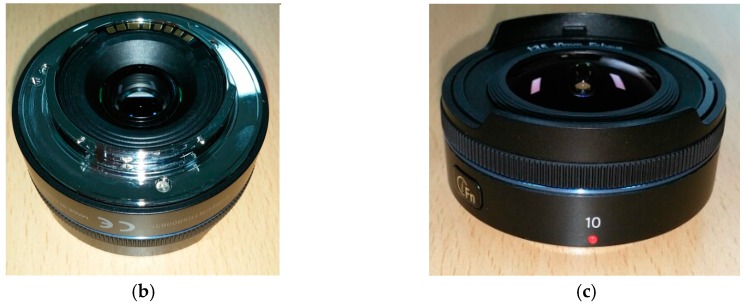
(**a**) Optical layout of the optimized fisheye lens system and (**b**) top and (**c**) bottom views of the fabricated fisheye lens system.

Even after the third-order aberrations have been corrected, the resolution of the designed fisheye lens system is not sufficient to enable characterization of the boundaries of illuminated targets, resulting in light being incident on surfaces not intended to be illuminated. Therefore, the optical design must be optimized using optical design software to correct additional aberrations. The lens design optimization process involves mathematically minimizing the spot size in the image plane by changing the curvature, thickness, material, aspherical coefficients, etc. of the fisheye lens [16]. Figure 3a depicts the optical layout of the optimized fisheye lens system, and Figure 3b,c are top and bottom views, respectively, of the manufactured fisheye lens system. The focusing processes, which compensate for changes in the image plane location, were performed considering the processing error compensation, assembly errors, position errors, and dependence of the focus on the position of the illuminated target. To perform these focusing processes, the entire fisheye lens system or the focusing lens needs to be moved to a certain position. By moving the focusing lens in this study, changes in the image plane location were corrected to focus the light, as shown in Figure 3. If other lenses are moved while the focusing lens is not, it is difficult for the fisheye lens system not to have a suitable resolution after focusing.

## 3. Results and Discussion

### 3.1. Fisheye Lens Performance Verification

**Figure 4:**
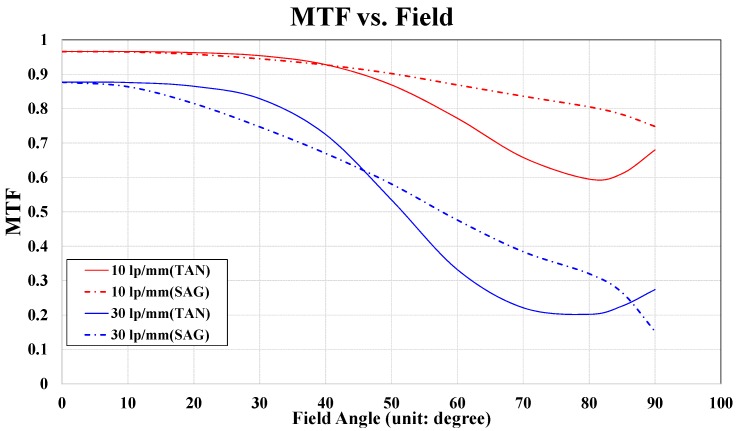
Field MTF plots for the optimized fisheye lens system (“lp” represents “line pair,” the units of spatial frequency).

When the designed fisheye lens is used for the photographic applications, we have to verify the MTF related with the resolving power. Optical lens companies provide MTF graphs instead of aberration and resolution data to demonstrate the excellent optical resolutions and aberrations of their lenses since aberration data are too extensive to be shown. MTF graphs cannot be obtained if the aberrations have not been compensated for. Figure 4 presents the MTF of the optimized fisheye lens system versus the field angle. The diagonal size of the image plane in Figure 3 was assumed to be 14.25 mm, similar to the sizes of the interchangeable lenses in a digital single lens reflex system. In a fisheye lens system with interchangeable lenses, the spatial frequency of the MTF is generally presumed to be 10 lp/mm or 30 lp/mm. Lens systems, such as fisheye lens systems, that include wide-angle lenses usually have MTFs of more than 0.2 at a spatial frequency of 30 lp/mm and at 0.8-field, which means 0.8 times the maximum field angle. These parameters yield a field angle of 72°. As shown in Figure 4, there are no optical performance problems because the MTF is greater than 0.2 when the field angle is 72°. In addition, a low MTF is acceptable, because the light illuminates the target. A low MTF indicates that the sharpness at the edges of the illumination surface is reduced, so more light can spread out into the fringe areas.

**Figure 5:**
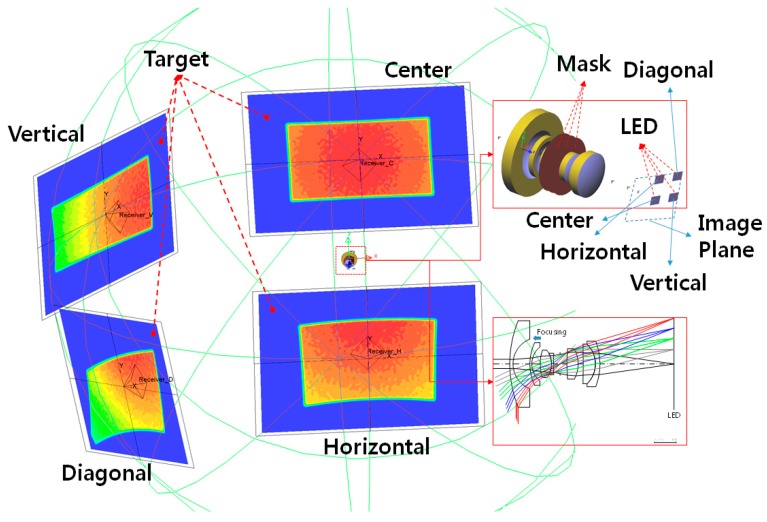
Fisheye lens illumination setup.

In the designed fisheye lens system, light-emitting diodes (LEDs) were utilized to illuminate the surface, enabling the light intensities and illumination distribution profiles to be checked. Figure 5 illustrates the setup used to obtain the illumination distribution profiles.

In Figure 5, the designed fisheye lens system is located in the center, and the details of the fisheye lens architecture are displayed on the right side. Four LED locations, called “Center,” “Horizontal,” “Vertical,” and “Diagonal,” were selected for the simulations. These locations represent the edges of the two-dimensional image plane. If the four edges of the image plane cover the target completely, the target can be viewed regardless of its location in the image plane. Thus, a target at any location could be illuminated in this type of fisheye lens system simply by moving the LEDs.

**Figure 6:**
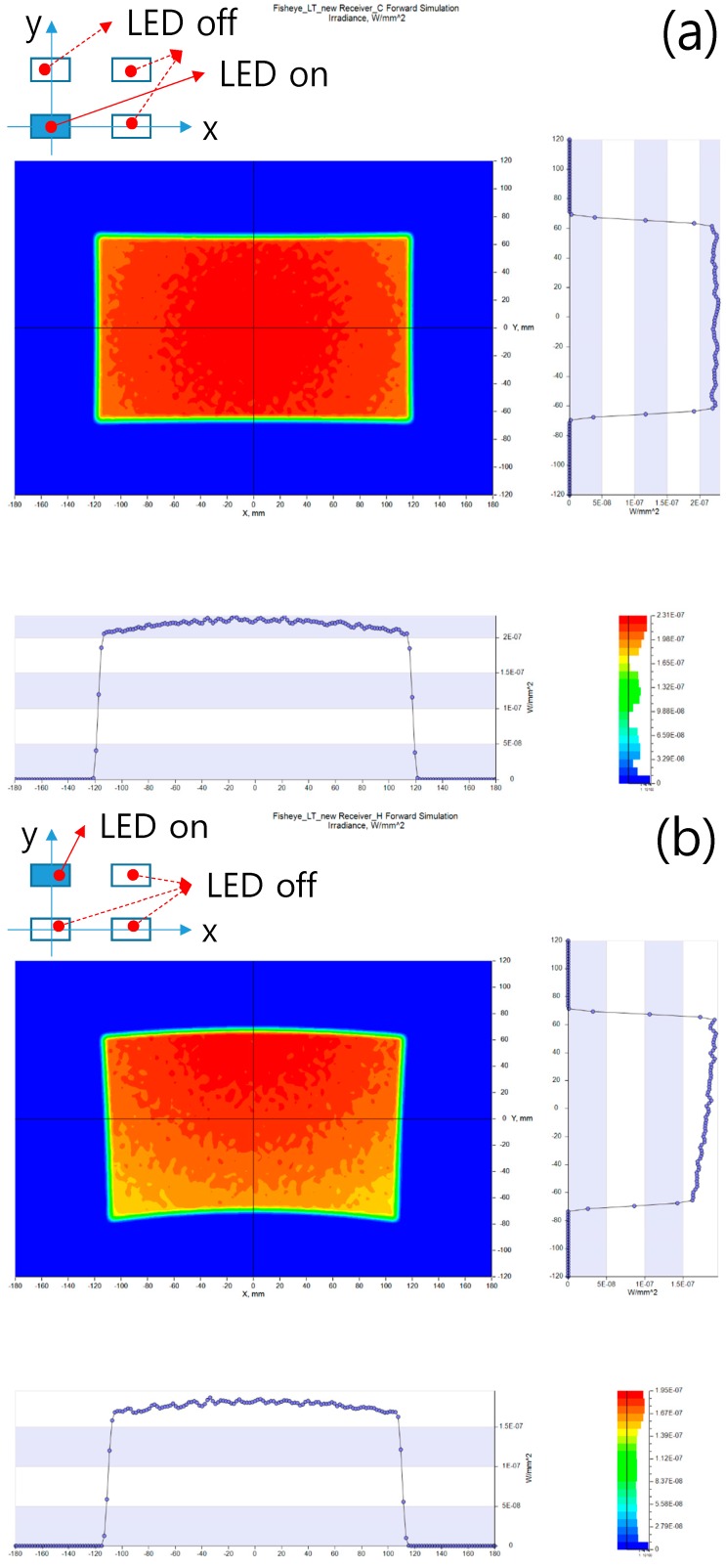

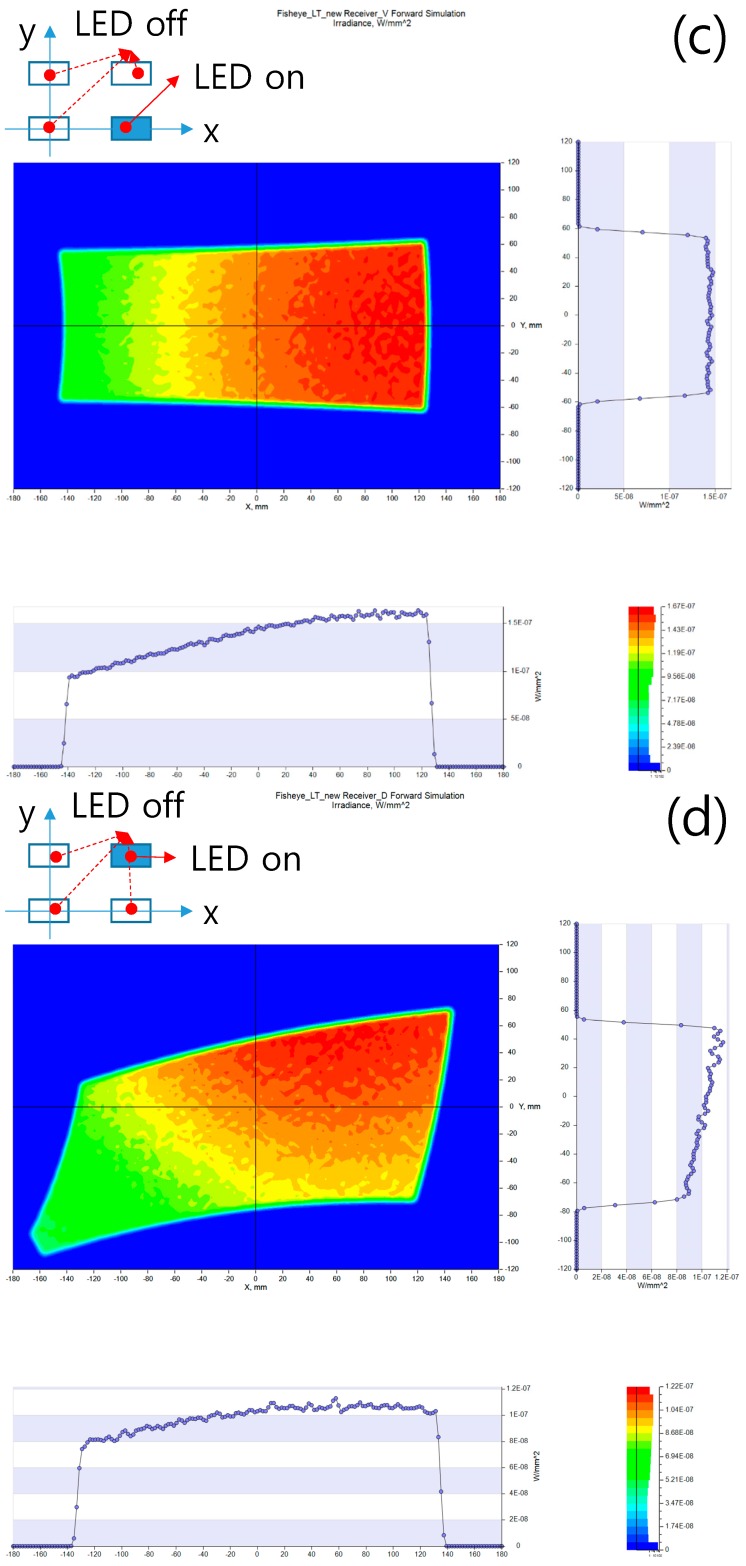
Intensity distributions on the targets obtained using the fisheye lens system. The light illumination profiles when LED light was placed in the (**a**) center, (**b**) horizontal, (**c**) vertical, and (**d**) diagonal positions.

Figure 6 presents detailed light intensity distribution profiles obtained at the four LED locations using electrical ON/OFF switches. To generate Figure 6a–d, the LEDs placed at the center of the system, +6.55 mm along the *y*-axis, +9.45 mm along the *x*-axis, and +9.45 mm in the *x*-direction and +6.55 mm in the *y*-direction, respectively, were used. The principal rays starting from the centers of the LEDs approached the illuminated surface perpendicularly, and the resulting intensity distributions are shown. Thus, the normal vector of the illuminated surface must match the directional vector of the principal ray.

The illuminated regions are not rectangular except in the case in which the LED was placed at the center of the image plane, due to the characteristics of the fisheye lens system. The system had a half-field angle of 90°, so the images of three-dimensional curved surfaces were transformed into two-dimensional surfaces. The light originating from the rectangular LED passed through the fisheye lens and was incident on the three-dimensional curved surfaces, so their shapes were accordingly changed into two-dimensional surfaces.

**Figure 7:**
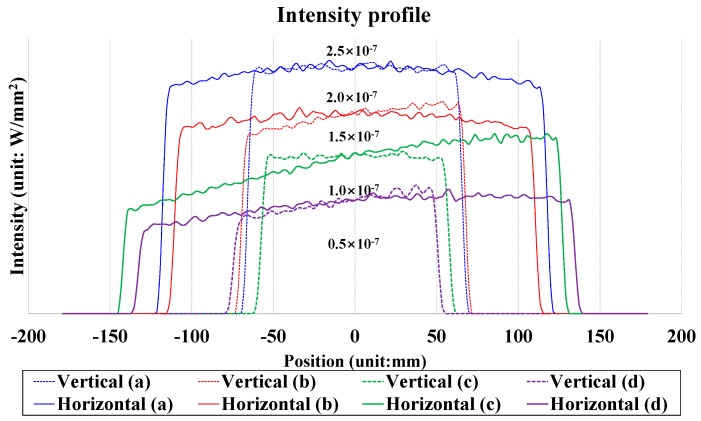
Intensity profiles corresponding to all four target locations and obtained using the fisheye lens system. The intensity profiles in the (**a**) vertical and horizontal directions when a LED light place was placed in the center position, intensity profiles in the (**b**) vertical and horizontal directions when a LED light place was placed in the horizontal position, intensity profiles in the (**c**) vertical and horizontal directions when a LED light place was placed in the vertical position, and intensity profiles in the (**d**) vertical and horizontal directions when a LED light place was placed in the diagonal position.

Figure 7 shows all of the light intensity distributions together. The solid and dotted curves represent the cross-sections of the intensity distributions in the horizontal and vertical directions, respectively. The horizontal and vertical axes indicate the light intensity and the LED position, respectively. The light intensity profiles on the illuminated surface are not uniform except in the case in which the LED was located at the center of the image plane. This behavior is evident because of the telecentric condition, i.e., because the telecentric condition, which means that the angle of the chief ray is 0° was not satisfied in order to reduce the fisheye lens size.

### 3.2. Pulse-Echo Responses

**Figure 8:**
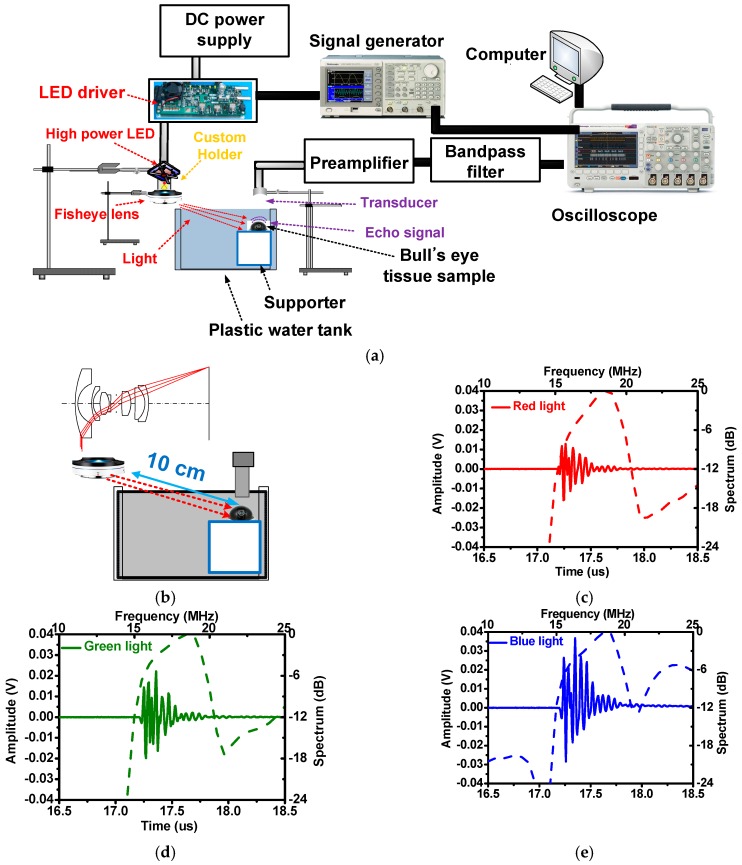
(**a**) Fisheye-lens-based PA system setup, (**b**) detailed setup of the LED and transducer, and the pulse-echo responses obtained using the (**c**) red, (**d**) green, and (**e**) blue LEDs.

Figure 8a depicts the experimental setup used to demonstrate the feasibility of the developed fisheye-lens-based PA system. A-mode pulse-echo response tests are typically performed to evaluate the performances of such systems [6,9,20,21]. The fisheye lens was located in the left corner slightly outside the water tank, and a transducer was immersed in the right portion of the water tank to acquire the echo signals from as far away as possible from the lens. A bull’s eye tissue sample was mounted on top of the support, separated diagonally by 10 cm from the arrival distance. Divergent pulsed high-power LED light sources (CBT-120, Luminus Devices, Sunnyvale, CA, USA) controlled by a LED driver (DK-136M-1, Luminus Devices, Sunnyvale, CA, USA) and a signal generator (AFG3252C, Tecktronics Inc., Beaverton, OR, USA) were used to transmit light into the fisheye lens and then onto the sample to cause rapid thermal expansion. To activate the high-power LEDs, 1 kHz, 3 V_p-p_, five-cycle narrowband pulsed waves produced by the signal generator were used to control the LED driver. The reflected ultrasonic signals caused by the thermal expansion were detected by a 20 MHz transducer with a focal length of 6 mm. Then, the signals were amplified by a 65 dB voltage preamplifier (AU-1525, L3 narda-MITEQ, Inc., Hauppauge, NY, USA) and a passive-type 20 MHz band-pass filter to enable the echo signals to be displayed on an oscilloscope (Tecktronics Inc.). After acquiring the echo signals, the spectral data were obtained using MATLAB (MathWorks, Natick, MA, USA). Figure 8b shows the configuration with the fisheye lens separated diagonally and vertically from the target. Figure 8c–e presents the spectra obtained when the lens was separated from the target diagonally. When red, green, and blue LED lights were used, the amplitudes were found to be 30.5 mV, 43.35 mV, and 67.04 mV; the center frequencies were determined to be 17.95 MHz, 17.75 MHz, and 17.71 MHz; and the −6 dB bandwidths of the echo waveforms were observed to be 15.04%, 24.67%, and 25.01%, respectively. The center frequencies of the echo signals were calculated by averaging the frequencies at the −6 dB bandwidth [21]. The amplitudes of the echo signals were different due to the different radiometric flux levels of red, green, and blue LED lights, according to the given datasheets.

**Figure 9:**
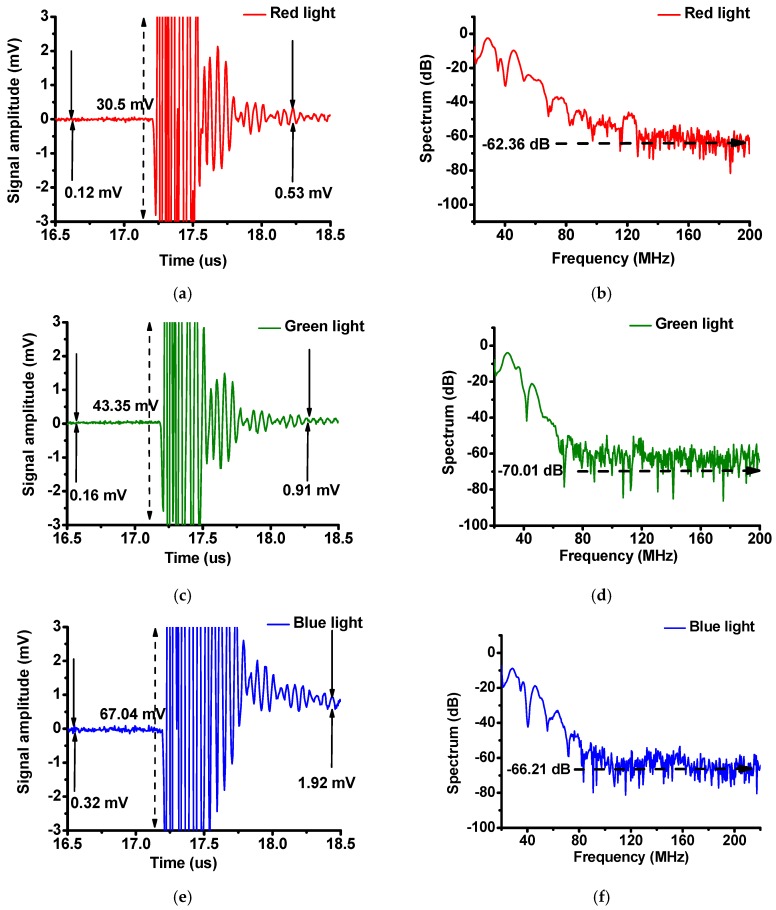
(**a**) Enlarged pulse echo responses and (**b**) their normalized spectra for the red LED lights, (**c**) enlarged pulse echo responses and (**d**) their normalized spectra for the green LED lights, and (**e**) enlarged pulse echo responses and (**f**) their normalized spectra for the blue LED light.

Figure 9 illustrates the noise levels in the time and frequency domains for the red, green, and blue LED lights. The noise levels in the time domain before the echo signals start and after they end are 0.12 mV and 0.53 mV, 0.16 mV and 0.91 mV, and 0.32 and 1.92 mV for the red, green, and blue LED lights, respectively. Meanwhile the noise levels in the frequency domain that were obtained using the red, green, and blue LED lights are −63.36 dB, −70.01 dB, and −66.21 dB, respectively. As shown in Figure 9, the echo signals are very clean and exhibit low noise in both the time and frequency domains since the voltages are zero before and after the echo signals in the time domain and the noise in the frequency domain is low. Therefore, these data prove that the proposed system can reduce the mechanical noise that occurs when using fisheye lens systems.

**Figure 10:**
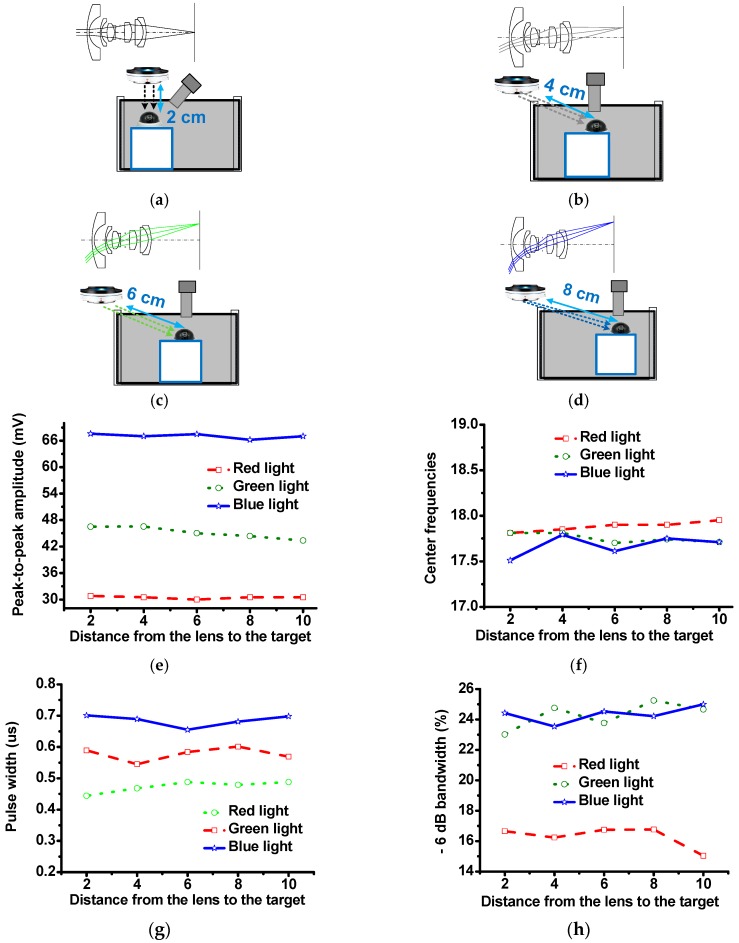
Configurations of the fisheye lens system and sample with the fisheye lens system (**a**) located 2 cm directly above the target and separated from the target diagonally by (**b**) 4 cm, (**c**) 6 cm, and (**d**) 8 cm. (**e**) Peak-to-peak echo amplitudes, (**f**) center frequencies, (**g**) pulse widths, and (**h**) −6 dB bandwidths vs. distance from the lens to the target. The results obtained using the red, green, and blue lights are represented by the dashed-dotted, dotted, and solid curves, respectively.

Figure 10 shows the configurations of the fisheye lens system, transducer, and sample that were used to verify the capabilities of the fisheye lens system, which can support a wide field of view. In Figure 10a, the fisheye lens system is located 2 cm directly above the target. In Figure 10b–d, the sample is separated diagonally from the fisheye lens system by 4 cm, 6 cm, and 8 cm, respectively, and LED light ray paths through the lens system are illustrated in the upper left corners of these figure parts. In Figure 10e, the peak-to-peak amplitudes of the echo waveforms acquired using red, green, and blue LED lights are 30.8, 30.5, 30 and 30.5 mV; 46.48, 46.5, 45, and 44.35 mV; and 67.60, 67.04, 67, and 66.21 mV, when the sample is located 2 cm directly below and separated diagonally by 4, 6, and 8 cm from the fisheye lens system, respectively. Figure 10g shows that the center frequencies measured using red, green, and blue LED lights are 17.81, 17.85, 17.9 and 17.9 MHz; 17.81, 17.8, 17.7, and 17.74 MHz; and 17.51, 17.79, 17.61, and 17.75 MHz. These measurements confirm that similar echo signal amplitudes and center frequencies could be obtained. In Figure 10g, the pulse widths measured using red, green, and blue LED lights are 0.444, 0.468, 0.488, 0.479 and 0.489 μs; 0.589, 0.545, 0.584, 0.601, and 0.569 μs; and 0.701, 0.678, 0.655, 0.681 and 0.698 μs, respectively. In Figure 10h, the −6 dB bandwidths measured using red, green, and blue LED lights are 16.66%, 16.24%, 16.75%, 16.76%, and 15.04%; 23.01%, 24.76%, 23.77%, 25.25%, and 24.67%; and 24.42%, 23.54%, 24.53%, 24.22%, and 25%, respectively. These measured pulse widths and −6 dB bandwidths also confirm that wide fields of view can be achieved using the fisheye lens system. The −6 dB bandwidths and pulse widths of the echo signals are related to the lateral and axial resolutions, respectively [6,22]. The measured results shown in Figure 10f,h demonstrates that the variances of the bandwidths and pulse widths are less than 10% and 3%, respectively. These results indicate that the fisheye lens system presented in this report can support a wide field of view.

## 4. Conclusions

This paper presents a novel fisheye-lens-based PA system capable of achieving a wide field of view with low abbreviations was presented. Therefore, this system can be possibly employed to reduce the effects of the mechanical motors that are used in conventional PA systems. Mechanical noises caused by motors affects the echo signal quality, and cannot be removed effectively during signal processing. In this study, LED light sources were used as a transmit source since LED light is a less harmful source than other types of light. However, the divergent characteristics of the LED light and mechanical noises require the use of specially designed lens systems, such as fisheye lens systems, that can focus the beams on certain locations efficiently. This is because the designed fisheye lens system can achieve a half-field angle of 90°, thereby significantly increasing the illuminated surface area and focusing the target information, even if the target moves relative to the light source.

To design the fisheye lens system in this study, one additional lens was used to enable the system to achieve a half-field angle of 90°. The focal length of the additional lens was selected to equal the difference between the focal lengths of the fisheye lens system and a conventional wide-angle lens system. During this selection process, the aspherical surface coefficient of our system was changed to compensate for third-order aberrations. Although the location of the image plane can be changed based on the object distance by moving the entire system, we designed the system to be of the inner-focus type, so that the overall system length would not change when focusing the image. In this manner, we designed a fisheye lens system capable of achieving a half-field angle of 90° and variable focusing based on the target distance. This lens system enabled the fabrication of a compact experimental PA system with electrical switches and without mechanical motors. Conventional fisheye lens systems, which include huge first lenses, cannot be integrated easily with electrical switches in PA systems.

The experimental results confirm that transducer could receive echo signals even when it was separated diagonally by 10 cm from the LED light. When red, green, and blue LEDs were used, the amplitudes were 30.8, 43.35 and 67.04 mV; the center frequencies were 17.95, 17.71 and 17.71 MHz; and the −6 dB bandwidths of the echo waveforms were 15.04%, 24.67%, and 25.01%, respectively. The variances of the bandwidths and pulse widths when the fisheye lens system was located 2 cm directly above and separated diagonally by 4, 6 and 8 cm from the sample were found to be less than 10% and 3%, respectively. The noise levels measured when using the red, green, and blue lights were −63.36, −70.01 and −66.21 dB, respectively. Therefore, we confirmed that the fisheye-lens-based PA system could be used to detect the echo signals even though the sample was separated from the LED light vertically or horizontally, thus providing wide fields of view with low noise. In the future, fisheye lens schemes and ultrasonic transducer arrays with advanced electronic scanning systems will be necessary to implement this PA system, which can completely remove the effects of mechanical noise (i.e., can obtain low-noise PA images) even though this method significantly increases the system cost and complexity. We believe that this kind of PA system could potentially impact even multidimensional ultrasound heart imaging, which requires low mechanical noise, because large target areas could be covered by the fisheye lens system, thereby reducing the effects of mechanical motor noise. We also expect the echo signal processing time to be reduced accordingly because the transmission source itself does not need to move from one position to another to scan the target completely.
